# Association of Glomerular Hyperfiltration and Cardiovascular Risk in Middle-Aged Healthy Individuals

**DOI:** 10.1001/jamanetworkopen.2020.2377

**Published:** 2020-04-10

**Authors:** Marie-Eve Dupuis, Annie-Claire Nadeau-Fredette, François Madore, Mohsen Agharazii, Rémi Goupil

**Affiliations:** 1 Research Centre of the Hôpital du Sacré-Cœur de Montréal, Department of Medicine, Université de Montréal, Montréal, Canada; 2 Research Centre of the Hôpital Maisonneuve-Rosemont, Department of Medicine, Université de Montréal, Montréal, Canada; 3 CHU de Québec, Hôtel-Dieu de Québec, Université Laval, Québec, Canada

## Abstract

**Importance:**

Glomerular hyperfiltration is associated with increased risk of cardiovascular disease in high-risk conditions, but its significance in low-risk individuals is uncertain.

**Objective:**

To determine whether glomerular hyperfiltration is associated with increased cardiovascular risk in healthy individuals.

**Design, Setting, and Participants:**

This was a prospective population-based cohort study, for which enrollment took place from August 2009 to October 2010, with follow-up available through March 31, 2016. Analysis of the data took place in October 2019. The cohort was composed of 9515 healthy individuals, defined as individuals without hypertension, diabetes, cardiovascular disease, estimated glomerular filtration rate (eGFR) less than 60 mL/min/1.73 m^2^, or statin and/or aspirin use, identified among 20 004 patients aged 40 to 69 years with health information accessed through the CARTaGENE research platform.

**Exposures:**

Individuals with glomerular hyperfiltration (eGFR >95th percentile after stratification for sex and age) were compared with individuals with normal filtration rate (eGFR 25th-75th percentiles).

**Main Outcomes and Measures:**

Adverse cardiovascular events were defined as a composite of cardiovascular mortality, myocardial infarction, unstable angina, heart failure, stroke, and transient ischemic attack. Risk of adverse cardiovascular events was assessed using Cox and fractional polynomial regressions and propensity score matching.

**Results:**

From the 20 004 CARTaGENE participants, 9515 healthy participants (4050 [42.6%] male; median [interquartile range] age, 50.4 [45.9-55.6] years) were identified. Among these, 473 had glomerular hyperfiltration (median [interquartile range] eGFR, 112 [107-115] mL/min/1.73 m^2^) and 4761 had a normal filtration rate (median [interquartile range] eGFR, 92 [87-97] mL/min/1.73 m^2^). Compared with the normal filtration rate, glomerular hyperfiltration was associated with an increased cardiovascular risk (hazard ratio, 1.88; 95% CI, 1.30-2.74; *P* = .001). Findings were similar with propensity score matching. The fractional polynomial regression showed that only the highest eGFR percentiles were associated with increased cardiovascular risk. The cardiovascular risk of individuals with glomerular hyperfiltration was similar to that of the 597 participants with an eGFR between 45 and 60 mL/min/1.73 m^2^ (hazard ratio, 0.90; 95% CI, 0.56-1.42; *P* = .64).

**Conclusions and Relevance:**

These findings suggest that glomerular hyperfiltration is independently associated with increased cardiovascular risk in middle-aged healthy individuals. This risk profile appears to be similar to stage 3a chronic kidney disease.

## Key Points

**Question:**

Is glomerular hyperfiltration associated with future cardiovascular events in healthy individuals?

**Findings:**

In this cohort study of 9515 patients with health information accessed through the CARTaGENE research platform, glomerular hyperfiltration was shown to be associated with increased risk of cardiovascular events in middle-aged healthy individuals.

**Meaning:**

This study found an increased cardiovascular disease risk associated with glomerular hyperfiltration in middle-aged healthy individuals, suggesting that glomerular hyperfiltration could be a useful cardiovascular biomarker in this population.

## Teaser

This population-based cohort study examines whether glomerular hyperfiltration is associated with a higher risk of cardiovascular events in healthy middle-aged individuals.

## Introduction

Chronic kidney disease (CKD), defined as an estimated glomerular filtration rate (eGFR) less than 60 mL/min/1.73 m^2^, is a widespread condition that affects millions of people throughout the world and is a well-recognized risk factor for cardiovascular morbidity and mortality.^1^ However, individuals with supranormal eGFR, or glomerular hyperfiltration (GHF), may also have an increased risk of cardiovascular diseases.^2,3,4^ Indeed, GHF may be viewed as a marker of vascular dysfunction in high-risk conditions such as diabetes, metabolic syndrome, hypertension, and disorders related to smoking, and it is associated with increased cardiovascular events.^5,6,7,8,9,10,11,12^ Interpretation of these studies is complex, as the definition of GHF is highly variable, from measured or eGFR cutoffs that are arbitrarily defined (90-175 mL/min/1.73 m^2^) to age- and/or sex-dependent definition, with or without consideration of ethnicity and concomitant antihypertensive treatment.^13^ Nevertheless, it is unknown whether GHF could also be associated with abnormal vascular dysfunction in an unfavorable metabolic milieu in apparently healthy individuals.

Therefore, the objective of this study was to characterize the cardiovascular risk associated with GHF in healthy individuals, using an epidemiologic definition of hyperfiltration with stratification for age and sex.^13^

## Methods

### Study Design, Population, and Measures

This study uses longitudinal follow-up data from the CARTaGENE populational cohort.^14^ This cohort, originally designed to investigate factors associated with chronic diseases, has been described elsewhere in detail.^15,16,17,18^ In brief, 20 004 individuals aged between 40 and 69 years were randomly selected in 4 metropolitan areas of the province of Quebec, Canada, between August 2009 and October 2010. This cohort is representative of the population of Quebec aged 40 to 69 years.^15^ Blood and urine samples were obtained from all participants. A full dietary habit questionnaire was completed by approximately half of CARTaGENE participants and used to estimate daily protein and sodium intake. Brachial blood pressure (BP) was measured after a 10-minute seated rest in an isolated room using an Omron 907L device (Omron); reported values are averages of 3 recordings. Central BP parameters were estimated during the same examination as brachial BP with a SphygmoCor PX device (Atcor Medical), using brachial systolic and diastolic BP for calibration of radial artery pressure wave.^18^ The following parameters were derived with the SphygmoCor device: (1) central systolic and diastolic BP; (2) central pulse pressure (PP); (3) PP amplification (brachial PP/central PP), which expresses the amplification ratio of the PP from central to peripheral arteries; (4) augmented pressure (central systolic BP − forward wave pressure), which represents the magnitude of the reflected wave; and (5) augmentation index ([augmented pressure/central PP] × 100), or the additional load to which the left ventricle is subjected because of backward wave reflection. The PP amplification, augmented pressure, and augmentation index can be considered indirect markers of arterial stiffness. Lean body mass was estimated with bioimpedance using a TBF-310 total body composition analyzer (Tanita). All participants provided written informed consent. This study adhered to the Declaration of Helsinki,^19^ was approved by the local ethics committees, and follows the Strengthening the Reporting of Observational Studies in Epidemiology (STROBE) reporting guidelines.^20^

### Definitions

Healthy individuals were defined by the absence of prior cardiovascular events (CVEs), diabetes, hypertension, eGFR less than 60 mL/min/1.73 m^2^ (stages 3-5 CKD^21^), or use of aspirin and/or statins. Prior CVEs were self-reported and included myocardial infarction, unstable angina, heart failure, stroke, and transient ischemic attack. Diabetes was defined as use of hypoglycemic agents and/or fasting glucose level greater than or equal to 126 mg/dL and/or nonfasting glucose level greater than or equal to 200 mg/dL (to convert to millimoles per liter, multiply by 0.0555) and/or hemoglobin A_1c_ level greater than or equal to 6.5% (to convert to proportion of total hemoglobin, multiply by 0.01).^22^ Hypertension was defined as use of any antihypertensive agents and/or BP greater than or equal to 140/90 mm Hg (mean of 3 recordings).

Healthy participants with GHF were defined by an eGFR greater than 95th percentile (or 2 SDs above the mean), and a normal glomerular filtration rate (control group) was defined as an eGFR between the 25th and 75th percentiles after stratification for age decade and sex.^5,12,13^ Glomerular filtration rates were estimated from serum creatinine measurements, calibrated by isotope dilution mass spectroscopy, using the Chronic Kidney Disease Epidemiology Collaboration (CKD-EPI) equation.^23^ Stage 3a CKD was defined as an eGFR between 45 and 60 mL/min/1.73 m^2^.^21^

### Outcome Measure

The primary outcome was CVE, defined as a composite of cardiovascular mortality, myocardial infarction, unstable angina, heart failure requiring hospitalization, stroke, and transient ischemic attack. Longitudinal follow-up data were obtained from databases of the Quebec health insurance agency Régie de l’assurance maladie du Quebéc (RAMQ), the Ministry of Health and Social Services (MED-ECHO), and the Institut de la Statistique du Québec. At the time of analysis, data were available from enrollment to March 31, 2016. As the Quebec government is the sole provider of health care in the province, data from all visits are available, including diagnosis codes and procedures during inpatient or outpatient encounters, discharge summaries, and the cause and date of death, with missing data only due to emigration from the province (<1%).^24^ These medicoadministrative databases and the guidelines for identifying cases established by the *International Statistical Classification of Diseases and Related Health Problems, Tenth Revision* have been previously validated for cardiovascular diagnosis.^25,26,27,28,29^

### Statistical Analysis

Normally distributed continuous data are presented as mean and standard deviation and were compared with *t* tests. Nonnormally distributed continuous data are presented as median (interquartile range [IQR]) and compared with Mann-Whitney tests. Categorical data were compared with Pearson χ^2^ tests. To evaluate the association between GHF and the first occurrence of CVE, a Cox proportional hazards model was developed with adjustment for covariables known or suspected to be associated with the outcome: age, sex, African American race, active smoking, body mass index, lean body mass, fasting glucose level, low-density lipoprotein cholesterol level, total cholesterol level, mean arterial pressure, and heart rate. This analysis was repeated using individuals with stage 3a CKD (eGFR of 45-60 mL/min/1.73 m^2^) as a control group, to compare the difference in CVE incidence between GHF and moderate CKD. The proportional hazards assumption was visually assessed with the log-minus-log plot.

Several sensitivity analyses were performed. First, GHF was also identified after stratification for age decade, sex, and (1) African American race, (2) active smoking, or (3) obesity (body mass index >30 [calculated as weight in kilograms divided by height in meters squared]). Second, to exclude a potential effect of ongoing subclinical disease processes already initiated before enrollment, washout periods of 6 and 12 months after enrollment were included. Third, the composite end point, CVE, was redefined without transient ischemic attack, as it is sometimes considered an outcome of lesser importance.

To further compare the difference between CVE occurrence between individuals with and without GHF, propensity score matching was performed, in an attempt to minimize confounding due to measured and unmeasured differences between the 2 groups.^30^ The propensity score was determined using a nonparsimonious logistic regression model, with GHF as the dependent variable and the covariables we have listed as independent variables. Using this propensity score, a nearest-neighbor 1:1 match without replacement was performed using caliper widths equal to 0.2 of the standard deviation.^31^ Appropriate balance between matched groups was acknowledged when absolute standardized differences were less than 10% for each of the covariables.^32^

To explore the nonlinear association between eGFR and CVE occurrence, a fractional polynomial regression model was used to determine the hazard ratios (HRs) of each 5% increment in eGFR percentile (after stratification for age decade and sex), with adjustment for the covariables listed. To compare potential differences in central BP profile, estimated marginal means (adjusted means) were calculated using general linear regression models, again adjusted for the above covariables (without brachial mean BP), and compared with post hoc Bonferroni-corrected univariate analysis of variance.

Multiple imputation was used to handle missing values (see eTable 1 in the Supplement for missing data list). Point estimates and their corresponding variances were combined according to Rubin rules.^33^ Two-sided *P* < .05 was considered significant. Analyses were performed with IBM SPSS Statistics (version 25.0; IBM Corp) and Stata IC (version 15.1; StataCorp) software.

## Results

Of the 20 004 CARTaGENE participants, 9515 (4050 [42.6%] male; median [interquartile range] age, 50.4 [45.9-55.6] years) met the criteria of healthy individuals. Among these, 473 participants had GHF (median [IQR] eGFR, 112 [107-115] mL/min/1.73 m^2^) and 4761 had a normal glomerular filtration rate (control group) (median [IQR] eGFR, 92 [87-97] mL/min/1.73 m^2^) (eFigure in the Supplement). Ranges of eGFR for individuals with GHF or normal filtration rates varied greatly according to each age decade and sex (eTable 2 in the Supplement). Participants with GHF were slightly younger, and more often African American or smokers (Table 1). Hemodynamic parameters were similar, apart from a slightly higher heart rate and augmentation index with GHF. Dietary habits were available in 213 individuals (45%) with GHF and 2387 individuals (50%) without GHF, which show similar median (IQR) daily protein (61 [36-86] vs 63 [37-89] g; *P* = .40) and sodium (2.2 [1.3-3.3] vs 2.3 [1.4-3.1] g; *P* = .80) intakes.

**Table 1.** Baseline Characteristics of Individuals With Normal Glomerular Filtration and Hyperfiltration

Characteristic	Normal glomerular filtration rate (n = 4761)	Glomerular hyperfiltration (n = 473)	*P* value
Age, median (IQR), y	51 (46-56)	50 (43-53)	<.001
Male, No. (%)	2027 (43)	201 (43)	>.99
African American, No. (%)	62 (1)	47 (10)	<.001
Body mass index^a^	26 (5)	26 (5)	.07
Lean body mass, mean (SD), kg	52 (11)	50 (11)	.03
Obesity, No. (%)^b^	763 (16)	78 (17)	.69
Active smoking, No. (%)	899 (19)	122 (26)	<.001
Estimated glomerular filtration rate, median (IQR), mL/min/1.73 m^2^	92 (87-97)	112 (107-115)	<.001
Framingham Risk Score, mean (SD), %	8 (7)	8 (7)	.53
Glucose, mean (SD), mg/dL	97 (18)	94 (18)	.003
Total cholesterol, mean (SD), mg/dL	205 (35)	197 (39)	<.001
Low-density lipoprotein cholesterol, mean (SD), mg/dL	124 (31)	120 (31)	.003
High-density lipoprotein cholesterol, mean (SD), mg/dL	50 (15)	50 (19)	.34
Systolic BP, mean (SD), mm Hg	118 (11)	118 (11)	.64
Diastolic BP, mean (SD), mm Hg	71 (8)	71 (9)	.86
Pulse pressure, mean (SD), mm Hg	47 (8)	46 (8)	.40
Heart rate, mean (SD), beats/min	67 (10)	69 (10)	.001
Central systolic BP, mean (SD), mm Hg	108 (11)	108 (11)	.95
Central diastolic BP, mean (SD), mm Hg	72 (8)	72 (9)	.77
Central pulse pressure, mean (SD), mm Hg	36 (8)	36 (7)	.67
Augmentation index, mean (SD), %	27 (11)	28 (11)	.04
Pulse pressure amplification, mean (SD)^c^	1.30 (0.15)	1.30 (0.14)	.36
Augmented pressure, mean (SD), mm Hg	10 (5)	10 (5)	.18
Systolic BP amplification, mean (SD), mm Hg	9 (4)	9 (4)	.27

Abbreviations: BP, blood pressure; IQR, interquartile range.

SI conversion factors: To convert glucose to mmol/L, multiply by 0.0555; cholesterol to mmol/L, multiply by 0.0259.

^a^ Calculated as weight in kilograms divided by height in meters squared.

^b^ Obesity is defined as presence of a body mass index greater than or equal to 30.

^c^ Pulse pressure amplification is determined by dividing brachial pulse pressure (in millimeters of mercury) by central pulse pressure (in millimeters of mercury).

### GHF and Cardiovascular Risk

**Figure 1.**
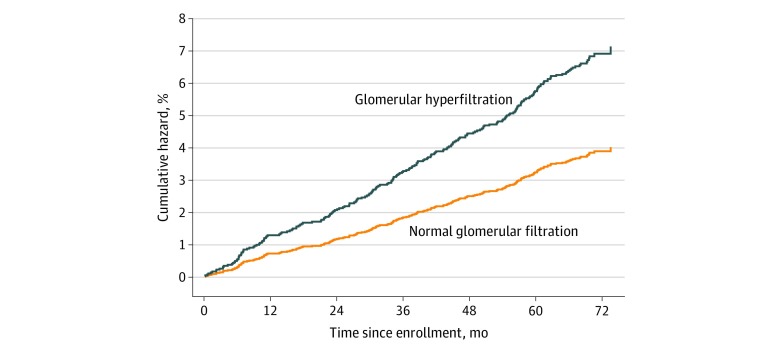
Cumulative Incidence of Adverse Cardiovascular Events in Individuals With Glomerular Hyperfiltration Compared With Normal Glomerular Filtration Adverse cardiovascular events are a composite of first onset of cardiovascular mortality, myocardial infarction, unstable angina, heart failure requiring hospitalization, stroke, and transient ischemic attack. Cox regression adjusted for age, sex, African American race, active smoking, body mass index, lean body mass, fasting glucose level, low-density lipoprotein cholesterol level, total cholesterol level, mean arterial pressure, and heart rate.

During a median follow-up of 70 months (IQR, 68-72), 245 CVEs occurred (3 cardiovascular deaths, 67 myocardial infarctions, 69 unstable angina episodes, 52 heart failure episodes, 34 strokes, and 20 transient ischemic attacks). In the GHF group, there were 35 events in 473 individuals (incidence rate of 13.2 per 100 person-years), whereas in the normal filtration group, there were 210 events in 4761 participants (incident rate of 7.7 per 100 person-years). In both the unadjusted and adjusted Cox regression analyses, GHF was associated with a higher risk of CVE compared with normal glomerular filtration rate (unadjusted HR, 1.71; 95% CI, 1.20-2.44; *P* = .003; adjusted HR, 1.88; 95% CI, 1.30-2.74; *P* = .001) (Table 2 and Figure 1). Glomerular hyperfiltration remained significantly associated with CVE in sensitivity analyses where GHF was defined after stratification for age decade, sex, and (1) race, (2) active smoking status, or (3) obesity (Table 2). Similarly, GHF remained significantly associated with CVEs after exclusion of participants with a CVE in the first 6 months (HR, 2.04; 95% CI, 1.39-2.99; *P* < .001) and 12 months (HR, 2.03; 95% CI, 1.35-3.06; *P* = .001), and after exclusion of transient ischemic attack from the composite outcome (HR, 2.03; 95% CI, 1.39-2.97; *P* < .001).

**Table 2.** Hazard Ratios for Cardiovascular Event Occurrence in the Primary, Secondary, and Sensitivity Analyses, With Various Ways to Determine Glomerular Hyperfiltration^a^

Analysis	Unadjusted models		Adjusted models^b^	
	Hazard ratio (95% CI)	*P* value	Hazard ratio (95% CI)	*P* value
Primary analysis				
GHF stratified by sex and age	1.71 (1.20-2.44)	.003	1.88 (1.30-2.74)	.001
Sensitivity analyses				
GHF stratified by sex, age, and race	1.62 (1.12-2.34)	.01	1.69 (1.15-2.46)	.008
GHF stratified by sex, age, and smoking status	1.56 (1.08-2.24)	.03	1.71 (1.04-2.80)	.03
GHF stratified by sex, age, and obesity	1.55 (1.06-2.26)	.02	1.59 (1.06-2.39)	.02
Propensity score matching analysis				
GHF stratified by sex and age	2.20 (1.44-4.24)	.02	NA	NA

Abbreviations: GHF, glomerular hyperfiltration; NA, not applicable.

^a^ Hazard ratios are calculated using Cox regression analyses and represent comparison with individuals with normal glomerular filtration.

^b^ Adjusted models included age, sex, African American race, active smoking, body mass index, lean body mass, fasting glucose level, low-density lipoprotein cholesterol level, total cholesterol level, mean arterial pressure, and heart rate. The adjusted model was not computed for the propensity score analysis as appropriate covariable balance was achieved.

**Figure 2.**
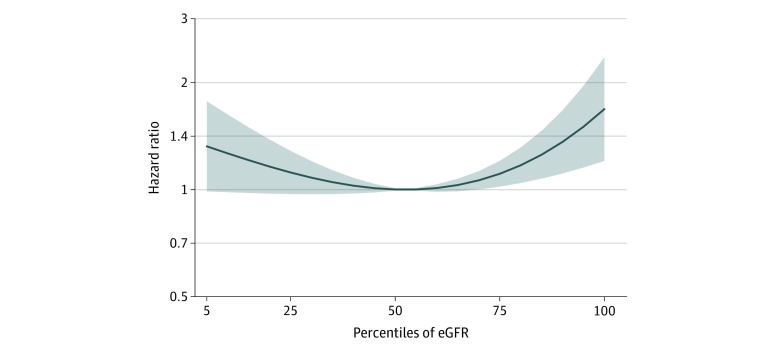
Fractional Polynomial Regression The graph represents the nonlinear association of estimated glomerular filtration rate (eGFR) with adverse cardiovascular event occurrence. Hazard ratios represent risk of cardiovascular events for each 5-percentile increase in age- and sex-stratified percentiles of eGFR. The hazard ratios become significantly greater than 1 above the 75th percentile. Adjusted for age, sex, African American race, active smoking, body mass index, lean body mass, fasting glucose level, low-density lipoprotein cholesterol level, total cholesterol level, mean arterial pressure, and heart rate. The black line represents the hazard ratio and the shaded area, the 95% confidence interval.

In the fractional polynomial regression analysis, a nonlinear association between CVE risk and eGFR percentiles was demonstrated, with a progressively higher risk association with eGFR above the 75th percentile (Figure 2).

By means of a propensity score, 406 healthy individuals with GHF were matched with 406 controls of similar age and baseline characteristics with appropriate covariate balance (eTable 3 in the Supplement). In this cohort, individuals with GHF had a median (IQR) eGFR of 112 (106-115) mL/min/1.73 m^2^ compared with 94 (88-99) mL/min/1.73 m^2^ in the control group. In an unadjusted Cox regression analysis, GHF was again associated with a higher CVE risk compared with normal filtration rate (HR, 2.20; 95% CI, 1.44-4.24; *P* = .02).

### Glomerular Hyperfiltration Compared With CKD

To examine the CVE risk associated with GHF compared with CKD, a subset of 597 CARTaGENE participants with stage 3a CKD were identified (eTable 4 in the Supplement). In an unadjusted Cox regression analysis, healthy individuals with GHF had a CVE risk similar to participants with stage 3a CKD (HR, 0.90; 95% CI, 0.56-1.42; *P* = .64), despite having a more favorable hemodynamic and metabolic profile and a lesser burden of comorbidities at baseline.

### Adjusted BP Parameters

Glomerular hyperfiltration was not associated with increased adjusted brachial or central systolic and diastolic BPs. It was, however, associated with marginally higher central PP and arterial wave reflection parameters (eTable 5 in the Supplement).

## Discussion

Using prospective data from the CARTaGENE cohort, this study demonstrates that GHF is associated with increased cardiovascular risk in healthy individuals compared with individuals with normal glomerular filtration rate. Of note, GHF was defined using an age- and sex-based epidemiological definition with a matched control group, as previously suggested.^13^ This allows identification of GHF using thresholds adjusted for sex and physiological decline of kidney function, avoiding arbitrarily selected thresholds. Interestingly, GHF was associated with a similar cardiovascular risk as stage 3a CKD, despite its lower burden of comorbidities.

Glomerular hyperfiltration has been shown in a multitude of studies to be associated with CVE incidence in populations with high-risk conditions such as obesity, diabetes, hypertension, and smoking.^2,3,4,5,6,7,8,9,10,11^ Unfortunately, various arbitrarily defined thresholds for the definition of GHF are used in a multitude of these studies, which may have introduced bias, as they can ignore sex-based differences and physiological age-related changes in eGFR.^13^ For example, data from the Chronic Kidney Disease Prognosis Consortium show an increased cardiovascular mortality in the presence of eGFR 105 mL/min/1.73 m^2^ or greater compared with a reference group with eGFR between 90 and 104 mL/min/1.73 m^2^.^4^ A previous study using the same population had shown that all-cause mortality increased at distinct eGFR thresholds in different age groups.^34^ How GHF leads to a worse cardiovascular prognosis may be, at least in part, due to maladaptive activation of the renin-angiotensin-aldosterone system, leading to altered systemic hemodynamic responses, endothelial dysfunction, and arterial stiffness.^35,36^ In addition, central BP may better indicate potential adverse cardiovascular outcomes compared with brachial BP,^37^ as it offers a more precise estimate of the true aortic BP, which directly affects central organs (heart, brain, and kidneys).^38^ In the present study, GHF was associated with increased arterial stiffness and wave reflection parameters. This observation is in keeping with the finding of Reboldi et al^5^ showing an increased PP in hypertensive individuals with GHF. Taken together, these findings suggest that arterial stiffness and increased pulse pressure transmission into the microcirculation could lead to microvascular damage and GHF.

However, it remained to be determined whether this increased cardiovascular risk associated with GHF is also present in healthy individuals. Recently, a large Korean registry study^2^ found a strong association between GHF and the risk of all-cause and cardiovascular mortality. Although the studied population was considered to be healthy, a high proportion of participants had elevated BP or diabetes and no data were provided regarding CVEs. Using a comparable definition of GHF to this study, Reboldi et al^5^ also found an increased cardiovascular risk of similar magnitude associated with GHF independent of ambulatory BP and albuminuria, but the study population was derived from an ambulatory BP registry and therefore included mostly individuals with hypertension. To our knowledge, the present study is the first to show that GHF is associated with cardiovascular risk in healthy individuals using a large, well-characterized North American cohort and previously validated medicoadministrative prospective data. Importantly, GHF was defined using epidemiological data based on serum creatinine level, an inexpensive, universally available biomarker, instead of arbitrary thresholds or cumbersome GFR measures, with the robustness of the definition demonstrated in sensitivity analyses.

The causes of GHF in the general population remain to be determined, but clues can be obtained from studies in physiological or pathological states such as pregnancy, diabetes, and obesity. As the GFR is determined by the renal plasma flow, the hydraulic pressure gradient across the glomerular basal membrane, and the ultrafiltration coefficient, alterations of any of these components can lead to GHF.^39^ In normal pregnancy, systemic vasodilation occurs with concomitant decrease in systemic vascular resistance, which, coupled with sodium retention, results in a reduction of resistance in both the afferent and efferent arterioles and ultimately leads to an increased renal blood flow and GHF.^39,40^ This appears to be mediated by relaxin, a hormone produced by the placenta that is responsible for increased release of nitric oxide and endothelin in renal microcirculation.^41^ Interestingly, GHF in pregnancy is not associated with long-term renal dysfunction, possibly as it is not associated with glomerular hypertension.^42^ On the other hand, GHF is one of the earliest manifestations of diabetic nephropathy. In contrast to pregnancy, diabetes leads to greater vasoconstriction of the efferent arteriole compared with the afferent arteriole, effectively causing GHF but also glomerular hypertension and an increase in the filtration fraction.^43^ Although the exact mechanisms remain uncertain, a multitude of mediators have been implicated, such as insulin-like growth factor 1, atrial natriuretic peptide, increased glucose and sodium retention, sorbitol, and advanced glycation products.^44,45,46,47^

Furthermore, individuals with obesity have higher eGFR than lean individuals.^48,49,50^ As in diabetes, altered renal hemodynamics lead to GHF with an increased filtration fraction. Activation of the renin-angiotensin-aldosterone system, through secretion of renin and renin precursors from adipocytes,^51^ appears to be the main mediator in obesity, although other mechanisms have been postulated.^49^ While GHF may be a good marker of generalized vascular dysfunction in patients with these conditions, and so might, therefore, be predictive of future cardiovascular events, it was surprising to see that even in healthy participants the presence of GHF could improve cardiovascular risk estimation. Therefore, it may be suggested that even in apparently healthy adults, GHF could represent a form of vascular dysfunction in an unfavorable neurohumoral milieu, one that cannot be identified by the traditional CV risk factors.

### Limitations

This study has several limitations. First, all CARTaGENE participants were randomly selected volunteers, which can introduce selection bias, although the sample appears to be an accurate representation of its source population.^15^ Also, only individuals aged 40 to 69 years were enrolled; therefore, generalization to older and younger individuals is uncertain. However, this population may yield the greatest benefit of early intervention. The GHF thresholds used in this study may also only be applicable to this cohort. Second, GFR was not measured but estimated using a single-serum creatinine value entered in the CKD-EPI equation.^23^ Nevertheless, this equation is validated in individuals with normal kidney function; it performs the best at higher eGFR but can still overestimate GFR, especially in presence of muscle wasting. In an attempt to reduce this potential bias, all analyses were adjusted for muscle mass estimated with bioimpedance. Also, eGFR cannot differentiate between GHF and a high number of nephron, a disorder that is thought to be physiological.^52^

However, the purpose of this study was not to accurately determine glomerular filtration rate but to assess the association of a universally available parameter with CVEs. Furthermore, if some of the individuals identified in this study as having GHF instead had a high number of nephron with normal single-nephron GFR, this would only strengthen our findings, as these individuals are not expected to have a higher cardiovascular risk. Third, data on albuminuria are only available in a very small subset of CARTaGENE participants and could not be considered in our analyses. In participants identified with GHF and normal filtration rate, only 33 (7%) and 248 (5%), respectively, had available albuminuria measurements. While conclusions with such small numbers are hazardous, the urine albumin to creatinine ratios were similar in both groups, and moderately increased albuminuria was present only in 5 individuals with a normal filtration rate and none with GHF. Fourth, although the study’s sample size was large, it was not sufficient to adequately assess the association between occurrence of individual types of CVEs and GHF.

## Conclusions

Using data from the prospective CARTaGENE cohort, GHF was shown to be associated with a higher risk of CVEs in healthy middle-aged individuals. Glomerular hyperfiltration could be an easily identifiable marker of an unfavorable metabolic milieu and vascular dysfunction. Therefore, identification of GHF in healthy individuals may provide an opportunity to implement preventive strategies to reduce the global burden of cardiovascular diseases.

## Notes

Supplement. **eTable 1.** Participants With Missing Data **eTable 2.** eGFR Range by Age Decade and Sex According to Glomerular Filtration Status **eTable 3.** Baseline Characteristics of Individuals Included in the Propensity Score-Matched Subgroup **eTable 4.** Baseline Characteristics of CARTaGENE Participants With Stage 3a CKD (eGFR Between 45-60 mL/min/1.73 m^2^) Compared to Subjects With Glomerular Hyperfiltration **eTable 5.** Adjusted Blood Pressure Parameters According to Glomerular Filtration Status **eFigure.** Study Design, Inclusions and Exclusions
