# Supplementary material for: Association of Glomerular Hyperfiltration and Cardiovascular Risk in Middle-Aged Healthy Individuals
Source: JAMA Netw Open. 2020 Apr 10;3(4):e202377. doi: 10.1001/jamanetworkopen.2020.2377 (PMC7148438; doi:10.1001/jamanetworkopen.2020.2377)
Supplement: Supplement. — eTable 1. Participants With Missing Data eTable 2. eGFR Range by Age Decade and Sex According to Glomerular Filtration Status eTable 3. Baseline Characteristics of Individuals Included in the Propensity Score-Matched Subgroup eTable 4. Baseline Characteristics of CARTaGENE Participants With Stage 3a CKD (eGFR Between 45-60 mL/min/1.73 m2) Compared to Subjects With Glomerular Hyperfiltration eTable 5. Adjusted Blood Pressure Parameters According to Glomerular Filtration Status eFigure. Study Design, Inclusions and Exclusions [file jamanetwopen-3-e202377-s001.pdf]

Supplementary Online Content

Dupuis M-E, Nadeau-Fredette A-C, Madore F, Agharazii M, Goupil R. Association of glomerular hyperfiltration and cardiovascular risk in middle-aged healthy individuals. *JAMA Netw Open*. 2020;3(4):e202377. doi:10.1001/jamanetworkopen.2020.2377

eTable 1. Participants With Missing Data
eTable 2. eGFR Range by Age Decade and Sex According to Glomerular Filtration Status
eTable 3. Baseline Characteristics of Individuals Included in the Propensity Score-Matched Subgroup
eTable 4. Baseline Characteristics of CARTaGENE Participants With Stage 3a CKD (eGFR Between 45-60 mL/min/1.73m^2) Compared to Subjects With Glomerular Hyperfiltration
eTable 5. Adjusted Blood Pressure Parameters According to Glomerular Filtration Status
eFigure. Study Design, Inclusions and Exclusions

This supplementary material has been provided by the authors to give readers additional information about their work.

© 2020 Dupuis M-E et al. *JAMA Network Open.*

eTable 1: Participants with missing data.

Missing data	Number of participants	percentage
Race	109	2.1%
Body mass index	39	0.7%
Lean body mass	166	3.2%
Smoking status	28	0.5%
Glucose	150	2.9%
Total cholesterol	129	2.5%

All other variables have no missing data.

© 2020 Dupuis M-E et al. *JAMA Network Open.*

eTable 2: eGFR range by age decade and sex according to glomerular filtration status.

Age group	Sex	Normal glomerular filtration rate		Glomerular Hyperfiltration	
		Number of participants	eGFR range	Number of participants	eGFR range
40-49 years	Male	1,015	85 to 104	101	111 to 190
	Female	1,252	85 to 105	125	112 to 128
50-59 years	Male	728	81 to 99	72	106 to 121
	Female	1,076	79 to 99	107	105 to 126
60-69 years	Male	284	75 to 93	28	98 to 105
	Female	406	75 to 93	40	99 to 114

Estimated glomerular filtration rates (eGFR) are estimated using the CKD-EPI equation and expressed as $mL/min/1.73m^2$.

© 2020 Dupuis M-E et al. *JAMA Network Open.*

eTable 3: Baseline characteristics of individuals included in the propensity score-matched subgroup.

Characteristics	Normal glomerular filtration rate (n=406)	Glomerular hyperfiltration (n=406)	Absolute standardized difference (%)
Age (years)	49 (46, 53)	50 (44, 54)	2.7
Male sex	178 (44%)	171 (42%)	3.4
Afro-American race	39 (10%)	38 (9%)	0.7
Body mass index (kg/m^2)	26 ± 6	26 ± 5	1.1
Lean body mass (kg)	51 ± 10	50 ± 11	5.3
Obesity	53 (13.1%)	65 (16%)	8.2
Smoking (active)	105 (26%)	107 (26%)	1.1
eGFR ($mL/min/1.73m^2$)	94 (88, 99)	112 (106, 115)	-
Framingham Risk Score (%)	7.2 ± 6.0	7.5 ± 6.9	4.8
Glucose (mg/dL)	5.2 ± 0.9	5.3 ± 1.0	2.4
Total cholesterol (mg/dL)	197 ± 35	197 ± 35	0.2
LDL-cholesterol (mg/dL)	120 ± 31	120 ± 31	0.5
HDL cholesterol (mg/dL)	50 ± 4	50 ± 19	3.9
Systolic BP (mmHg)	117 ± 12	118 ± 11	0.2
Diastolic BP (mmHg)	71 ± 9	71 ± 9	5.0
Heart rate (bpm)	68 ± 11	68 ± 10	2.7

Values are expressed mean ± standard deviation or median (25th, 75th percentiles) as appropriate. Matching variables include age, sex, Afro-American race, active smoking, body mass index, lean-body mass, fasting glucose, LDL-cholesterol, total cholesterol, mean arterial pressure and heart rate. Absolute standardized differences < 10% indicate adequate balance. Abbreviations as defined in Supplemental Table 1.

© 2020 Dupuis M-E et al. *JAMA Network Open.*

eTable 4: Baseline characteristics of CARTaGENE participants with stage 3a CKD (eGFR between 45-60 mL/min/1.73m^2) compared to subjects with glomerular hyperfiltration.

**Characteristics**	**Stage 3a CKD (n=597)**	**Glomerular hyperfiltration (n=473)**	***P* value**
Age (years)	63 (56, 67)	50 (43, 53)	<0.001
Male sex	290 (49%)	201 (43%)	0.05
Afro-American race	9 (2%)	47 (10%)	<0.001
Body mass index (kg/m^2)	29 ± 6	26 ± 5	<0.001
Lean body mass (kg)	53 ± 12	50 ± 11	<0.001
Diabetes	113 (12%)	0 (0%)	<0.001
Past cardiovascular disease	73 (19%)	0 (0%)	<0.001
Treated hypertension	286 (48%)	0 (0%)	<0.001
Obesity	204 (34%)	78 (17%)	<0.001
Smoking (active)	81 (14%)	122 (26%)	<0.001
eGFR (mL/min/1.73m^2)	56 (52, 58)	112 (107, 115)	<0.001
Framingham Risk Score (%)	18 ± 15	8 ± 7	<0.001
Glucose (mg/dL)	112 ± 41	94 ± 18	<0.001
Total cholesterol (mg/dL)	193 ± 46	197 ± 39	0.2
LDL-cholesterol (mg/dL)	112 ± 39	120 ± 31	0.001
HDL cholesterol (mg/dL)	46 ± 15	50 ± 19	<0.001
Systolic BP (mmHg)	126 ± 16	118 ± 11	<0.001
Diastolic BP (mmHg)	72 ± 10	71 ± 9	0.8
Heart rate (bpm)	69 ± 12	69 ± 10	1.0
Aspirin	154 (26%)	0 (0%)	<0.001
Statins	195 (33%)	0 (0%)	<0.001
Renin-angiotensin blockers	222 (37%)	0 (0%)	<0.001
Calcium-channel blockers	85 (14%)	0 (0%)	<0.001
Beta-blockers	92 (15%)	0 (0%)	<0.001
Diuretics	112 (19%)	0 (0%)	<0.001

Values are expressed as mean ± standard deviation or median (25th ,75th percentiles). To convert glucose and cholesterol to SI units (mmol/L), divide by 18 and 38.7. eGFR, estimated glomerular filtration rate; LDL, low-density lipoprotein; HDL, high-density lipoprotein; BP, blood pressure.

© 2020 Dupuis M-E et al. *JAMA Network Open.*

eTable 5: Adjusted blood pressure parameters according to glomerular filtration status.

Adjusted BP parameters	Normal glomerular filtration rate	Glomerular Hyperfiltration	p-value
Systolic BP (mmHg)	119 (118-120)	119 (118-121)	0.3
Diastolic BP (mmHg)	72 (71-73)	72 (71-73)	1.0
Pulse pressure (mmHg)	47 (46-48)	47 (47-48)	0.1
Central systolic BP (mmHg)	110 (109-111)	110 (109-112)	0.2
Central diastolic BP (mmHg)	73 (72-74)	73 (72-74)	1.0
Central pulse pressure (mmHg)	37 (36-37)	38 (37-38)	0.02
Augmentation index (%)	28 (27-29)	29 (28-30)	0.001
Pulse pressure amplification	1.29 (1.28-1.30)	1.28 (1.26-1.29)	0.007
Augmented pressure (mmHg)	10 (10-11)	11 (11-12)	0.001
BP amplification (mmHg)	9.1 (8.8-9.5)	9.0 (8.5-9.3)	0.3

Estimated marginal means (95% confidence interval) adjusted for age, sex, Afro-American race, active smoking, body mass index, lean-body mass, fasting glucose, LDL-cholesterol, total cholesterol, mean arterial pressure and heart rate. BP, blood pressure.

© 2020 Dupuis M-E et al. *JAMA Network Open.*

eFigure: Study design, inclusions and exclusions.

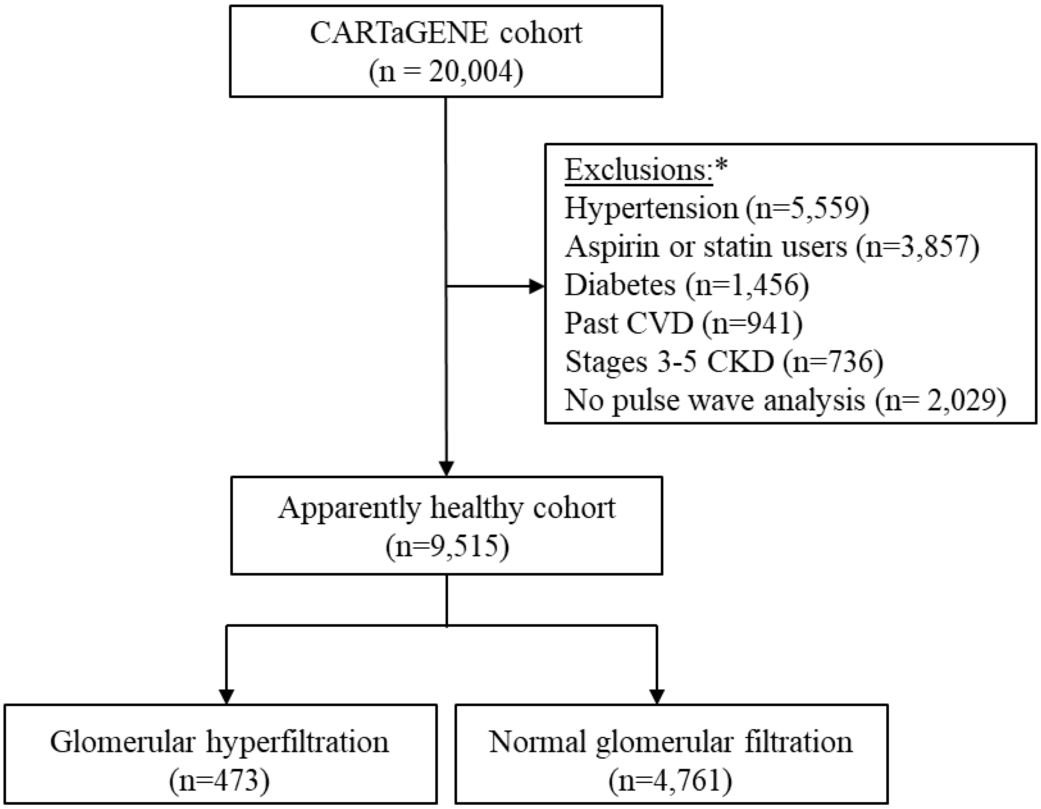

Glomerular hyperfiltration defined as an eGFR > 95th percentile after stratification for age decade and sex and normal glomerular filtration as an eGFR between the 25th and 75th percentiles. Participants with stage 3a CKD (n=597) were used as a secondary comparator group, irrespective of the presence of other excluded comorbidities.
* some individuals have more than one exclusion.

© 2020 Dupuis M-E et al. *JAMA Network Open.*
